# Meta-analysis and systematic review of amiodarone-inclusive treatment for fetal tachyarrhythmia

**DOI:** 10.3389/fphar.2026.1912369

**Published:** 2026-09-07

**Authors:** Lingjun Kong, Yanan He, Xiang Li, Yuan Lu, Yahui Zhang

**Affiliations:** 1 Department of Pharmacy, Shandong Provincial Hospital Affiliated to Shandong First Medical University, Jinan, Shandong, China; 2 Gamma Knife Center, Shandong Provincial Hospital Affiliated to Shandong First Medical University, Jinan, Shandong, China; 3 School of Pharmacy, China Pharmaceutical University, Nanjing, Jiangsu, China; 4 Department of Obstetrics and Gynaecology, Shandong Provincial Hospital Affiliated to Shandong First Medical University, Jinan, Shandong, China

**Keywords:** amiodarone, fetal tachyarrhythmia, hydropic, management, thyroid dysfunction

## Abstract

**Objective:**

To analyze the efficacy of amiodarone-inclusive protocol and the safety of amiodarone in fetal tachyarrhythmia treatment.

**Methods:**

PubMed, Web of Science, Medline and Embase were searched. Randomized controlled trials, cohort studies, case series and case reports on the efficacy of amiodarone-based therapy and the amiodarone-related safety in fetal tachycardia were included. Case reports were presented separately and descriptively synthesized, while case series and cohort studies were included in the meta-analysis. Outcomes included conversion of the fetal rhythm and the occurrence of adverse effects induced by amiodarone in the fetus. This research was registered in PROSPERO (No.CRD42024560291).

**Results:**

We analyzed 107 pregnancies treated with amiodarone from 27 studies. A descriptive synthesis of 18 case reports yielded a cardioversion rate of 81.0% for amiodarone-inclusive protocol, which was further supported by a quantitative analysis of nine studies showing a pooled cardioversion rate of 69.9% (95% CI: 57.6%–81.2%). In the hydropic subgroup fetus, cardioversion rates ranged from 53.8% to 87.5% (median, 63.4%). Overall, adverse events were reported in 29 fetuses, with thyroid dysfunction being the most prevalent. The pooled incidence of thyroid dysfunction was 21.2% (95% CI:11.4%–32.6%).

**Conclusion:**

Amiodarone-inclusive protocol may be effective in fetal tachycardia management, particularly in refractory cases. These findings support amiodarone as an adjunctive therapy rather than monotherapy. Although its use was related to a risk of transient neonatal thyroid dysfunction, there was insufficient data to support an increase in the severe adverse reactions.

**Systematic Review Resgistration:**

PROSPERO (No. CRD42024560291).

## Introduction

Fetal tachyarrhythmia is defined as cardiac rhythms or heart rates greater than 180 beats per minute in fetuses and occurs in approximately 0.1% of pregnancies (Tamirisa et al., 2022). It mainly manifests as supraventricular tachycardia (SVT), atrial flutter (AF), or ventricular tachycardia (Carvalho, 2019). While most cases of fetal tachyarrhythmias are benign and transient, persistent tachyarrhythmias can result in secondary damage to vital organs and are often associated with fetal hydrops and heart failure, as well as premature birth and death. Hydrops fetalis is observed in 30%–40% of fetuses with SVT and in 7%–43% of fetuses with AF, a condition relating to adverse outcomes during pregnancy (Miyoshi et al., 2019).

Transplacental antiarrhythmic therapy via maternal administration is strongly recommended for sustained tachyarrhythmia. According to the AHA guidelines on fetal tachycardia, first-line pharmacotherapy includes digoxin, flecainide, and sotalol. Digoxin has a low and unpredictable transplacental passage rate, and its efficacy is markedly reduced in the presence of hydrops fetalis. Flecainide and sotalol have limitations in the treatment of refractory or life-threatening fetal tachyarrhythmias (Donofrio et al., 2014). When first-line agents are ineffective or severe fetal hydrops develops, alternative treatments are urgently needed. Amiodarone, a broad-spectrum antiarrhythmic agent with Class I, II, III, and IV channel-blocking activity, plays a central role in the treatment of various cardiac arrhythmias, particularly shock-refractory ventricular fibrillation (VF) or pulseless ventricular tachycardia (VT). Several studies have mentioned the potential efficacy of amiodarone in the treatment of fetal tachyarrhythmia, particularly in cases complicated by hydrops fetalis or refractory arrhythmia (Donofrio et al., 2014; Jouannic et al., 2002; Strasburger et al., 2004). The American Heart Association recommends the use of amiodarone as a third-line therapeutic agent during pregnancy, predominantly owing to its association with an increased risk of adverse reactions (Batra et al., 2024). Nevertheless, the studies referred to are predominantly characterized by small sample sizes and single-center designs (Batra et al., 2024; Alsaied et al., 2017). The objective of our systematic review and meta-analysis is to investigate the effectiveness of amiodarone-containing regimen and the safety of amiodarone in the treatment of fetal tachyarrhythmia, thereby providing more evidence on intrauterine medical therapy for fetal tachyarrhythmia.

## Materials and methods

### Study protocol and registration

The systematic review was performed according to the preferred reporting items for systematic reviews and meta-analyses (PRISMA) (Page et al., 2021). It followed a registered protocol that was developed *a priori* and registered (PROSPERO: CRD42024560291).

### Data sources and search strategy

With the collaboration of the study’s lead investigator, an experienced librarian developed and implemented the search strategy. Studies were searched in the databases of PubMed, Web of Science, Medline and Embase by search terms of ((Tachyarrhythmia OR Tachycardia OR Tachycardia Supraventricular OR flutter) AND (fetal OR fetus) AND (Anti-Arrhythmia Agents OR Amiodarone)). We also reviewed the reference lists for recent reviews, editorials, and pertinent publications.

### Selection of studies

In the absence of randomized controlled trials on the treatment of fetal tachyarrhythmia, we focused on retrospective and prospective cohort studies, case series, and case reports that reported amiodarone-containing treatment for fetal tachyarrhythmia. There were no restrictions on the sample size. A comprehensive search of multiple databases was conducted from the inception of each database until 23 March 2026. Two authors independently performed the literature search and conducted data extraction using a standardized, prespecified form that distinguished the types of tachycardia or whether hydrops existed. Inclusion criteria: (1) patients with fetal tachyarrhythmia, or patients with non-sustained fetal tachycardia with evidence of cardiac dysfunction and/or hydrops treated with amiodarone will be included in the analysis regardless of their ethnicity and background. (2) We defined amiodarone treatment as an experimental intervention, regardless of the treatment route. The efficacy of combinations of amiodarone-inclusive protocol and other drugs was also taken into account. (3) The primary outcome was the effect of amiodarone therapy, defined as the conversion of fetal rhythm to sinus rhythm (SR) or rate control in fetuses suffering from tachyarrhythmia. Secondary outcomes considered were the adverse effects of amiodarone on fetuses. Publications that fulfilled any of the subsequent requirements were excluded: (1) Patients with immunologic or non-sustained fetal tachyarrhythmias. (2) Research on outcomes not directly related to our specified primary outcomes. (3) Duplicate publications. (4) The search was not the English-language publications.

### Data extraction

The identification and extraction of potentially eligible articles were conducted by two researchers independently. A third reviewer was invited to resolve any discrepancies. Subsequently, a comprehensive analysis of their full texts was performed after the identified articles were retrieved. The following study characteristics included: general information of the study (author, publication year, region), population (number, diagnosis), study design, amiodarone intervention (dosage, administration, duration), outcomes (conversion to SR, rate control, incidence of adverse events). In cases where these data were not available, calculations were made following the details provided within the articles.

### Quality assessment

No randomized trials have been conducted on fetal tachycardia treated with amiodarone. The case series study were evaluated utilizing the Methodological Index for Non-Randomized Studies (MINORS). The MINORS score, which ranged from 0 to 16, consists of eight components. A 3-point rating system (0–2) was used for each item. The quality of studies was categorized as “high” (score of 13–16), “medium” (score of 9–12), or “low” (score of less than 8) (Yang et al., 2025). Case reports were evaluated using the Joanna Briggs Institute (JBI) Critical Appraisal Checklist for Case Reports (8 items, score range, 0–8). Scores of 7–8 indicated high quality, 5–6 moderate quality, and less than 4 as low quality (Peters et al., 2017).

### Risk of bias

For the descriptive synthesis of case reports, formal assessment of publication bias was not undertaken. Case reports are subject to high selection bias, as clinicians selectively record and submit unusual or favorable outcomes. The included case reports were performed as the quantitative synthesis rather than pooled effect estimates, and they were evaluated for data completeness rather than methodological rigor.

For the pooled analysis of nine single-arm studies, publication bias could not be assessed due to having fewer than ten studies and the lack of control groups. Evidence robustness was instead determined through systematically eliminating each study. Publication bias was evaluated using a funnel plot of the overall cardioversion rate. Funnel-plot asymmetry was analyzed qualitatively. Although any publication bias could not be completely ruled out, the symmetry was demonstrated to indicate the lack of a significant small-study effect. No Egger or Begg test was performed because of insufficient papers in small-study sets, in accordance with the Cochrane Handbook.

### Data synthesis and analysis

We have divided the analysis into two distinct types. Certain outcomes permitted a pooled quantitative meta-analysis, while other variables lacked enough data for statistical pooling were summarized descriptively. Therefore, the case reports were summarized in tables and synthesized qualitatively. No quantitative pooling was performed for the section of case reports. In contrast, case series or retrospective studies were quantitatively analyzed using meta-analysis with R Studio software. The pooled proportion of cardioversion and adverse events were calculated separately, along with their 95% confidence intervals. The Freeman-Tukey double arcsine transformation was applied to stabilize the variance and compute reliable confidence intervals. A random-effects model was adopted to account for potential clinical heterogeneity across the included studies. In addition, subgroup analyses by hydrops, route of administration, and amiodarone monotherapy versus combination management were presented descriptively due to the limited number of studies and incomplete reporting data for formal meta‐analytic pooling.

## Results

### Characteristics of the studies

**FIGURE 1:**
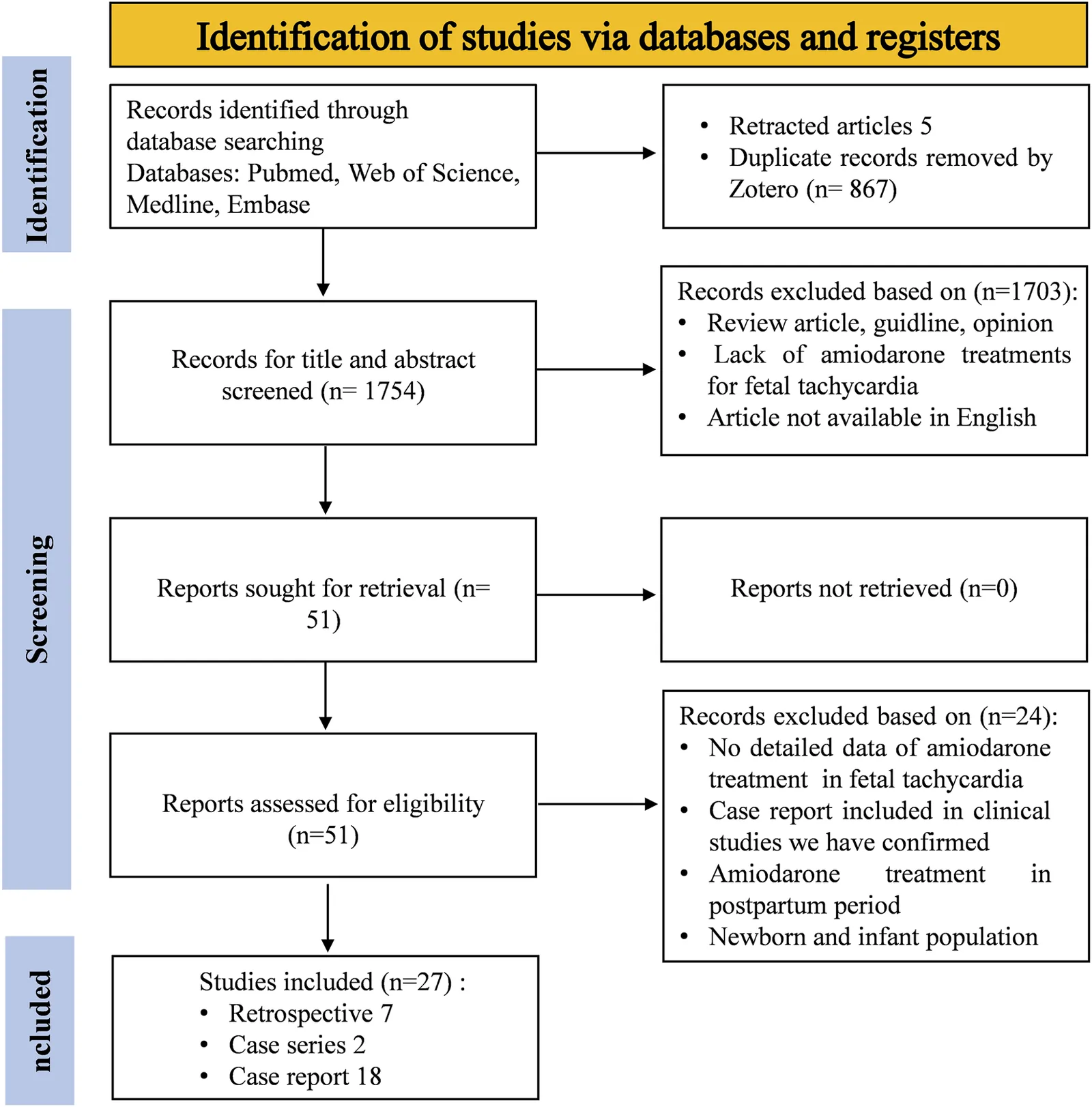
PRISMA for systematic reviews and meta-analyses flow diagram for study selection.

A total of 2,626 relevant articles were initially discovered. Titles and abstracts were screened for 1,759 records subsequent to the elimination of duplicates and retracted articles. Then, 51 full texts articles were evaluated for detailed information, of which 27 fulfilled the eligibility requirements (Figure 1) (Jouannic et al., 2002; Strasburger et al., 2004; Adenin and Kapnosa Hasani, 2023; Huang and Wolf, 2019; Minisha et al., 2019; Lin et al., 2016; Capone et al., 2014; Pradhan et al., 2006; Khositseth et al., 2003; Vanbesien et al., 2001; Schleich et al., 2000; Mangione et al., 1999; Lupoglazoff et al., 1999; De Catte et al., 1994; Jouppila et al., 1993; Flack et al., 1993; Gembruch et al., 1989; Arnoux et al., 1987; Laurent et al., 1987; Rey et al., 1985; Moatassim et al., 2018; Kang et al., 2015; Pézard et al., 2008; Jouannic et al., 2003; Simpson and Sharland, 1998; Azancot-Benisty et al., 1992; Hansmann et al., 1991), including seven retrospective studies, two case series, and 18 case reports. However, after methodological assessment, none of the retrospective studies included a comparator group, and they were reclassified as case series in the final analysis. Of these, 18 case report were synthesized descriptively. The remaining nine case series were eligible for the quantitative meta-analysis.

### Characteristics of patients

Table 1 presents the characteristics of fetuses receiving amiodarone monotherapy or combination therapy, as reported in the 27 included studies. A total of 107 patients were enrolled, of whom 21 were from case reports, 86 from the nine studies included in meta (Table 1). Considering medication, only 24 of 107 pregnancies with fetal tachyarrhythmias were treated with amiodarone as first-line therapy. Digoxin was the most frequently used antiarrhythmic drug, accounting for approximately 72.9% (78/107) of the total, regardless of whether it was the first-line treatment. Various antiarrhythmic drugs, such as flecainide, sotalol, and verapamil, were commonly used in these studies. There was also involvement with other classical antiarrhythmic drugs, such as procainamide, propafenone, atenolol, and propranolol.

**TABLE 1:** Summary of clinical characteristics (derived from case reports, case series).

Author	Year	Study design	Pregnancies treated with amiodarone								
			Sample size	Mechanism	Hydrops (patient no.)	First line?	Other anti-arrhythmic drugs	Dosage and route	Exposure time	Therapeutic response	Adverse events of fetus
Adenin and Kapnosa Hasani (2023)	2023	Case report	1	SVT	1	Yes	​	Oral: 100–1,200 mg IV: 2.5 mg/kg IP: 2.5 mg/kg	3 weeks	Control	No side effects
Huang and Wolf (2019)	2019	Case report	1	SVT	1	No	Digoxin Sotalol Flecainide	Oral: 400–800 mg/d	10 days	Improved	Transient hypothyroidism
Minisha et al. (2019)	2018	Case report	1	SVT	1	No	Digoxin Flecainide Sotalol	Oral: loading 2,400 mg/d (800 mg 3 times a day)	2 weeks	Control	No side effects
Lin et al. (2016)	2016	Case report	1	AF	1	Yes	Digoxin	IP + IV: 75–300 mg	3 weeks	Control	No side effects
Capone et al. (2014)	2014	Case report	1	SVT	1	No	Digoxin Flecainide	Oral: 600–2400 mg/d IV: 5 mg	14 days (IV four times)	Control	Hypothyroidism
Pradhan et al. (2006)	2006	Case report	1	SVT	1	No	Digoxin	Oral:1,000 mg/day for 2 days followed by 400 mg/d	5 weeks (32–37 weeks)	Control	No side effects
Khositseth et al. (2003)	2003	Case report	3	SVT 2 AF 1	3	Yes 1 No 2	Digoxin (3) Procainamide (1) Propranolol (1) Adenosine (1)	Case 1: oral 400–1,600 mg/ d Case 2: oral (loadng dose 150 mg) +IV Case 3: oral 400 mg three times a day	Case 1: 78 days Case 2: 21 days Case 3: 13 days	Control 3	Hypothyroidism: 1 No side effects 2
Vanbesien et al. (2001)	2001	Case report	2	SVT	2	Yes 0 No 2	Digoxin (2) Flecainide (1) Propafenone (1)	Oral:200 mg/d IV:2.5–5 mg once	Case 1: 1 week Case 2: 6 weeks	Control 2	TSH increased: 2
Schleich et al. (2000)	2000	Case report	1	SVT	1	No	Digoxin Sotalol Propranolol	Oral: 200–1,000 mg/d	5 weeks	Control	No side effects
Mangione et al. (1999)	1999	Case report	1	SVT	1	No	Digoxin Fecainide	IV: 10 mg Oral: 2 g/d	5 days + 3 times injection	Control	T4 level abnormal
Lupoglazoff et al. (1999)	1999	Case report	1	JET (familial history)	1	Yes	​	Oral: 400–800 mg/d	1 week	Improved	Not mentioned
De Catte et al. (1994)	1994	Case report	1	SVT	1	No	Digoxin Propafenone	IV: 2.5–5.5 mg	8 times injection	Control	No goitre, mild hypothyroidism
Jouppila et al. (1993)	1993	Case report	1	SVT	1	No	Digoxin Atenolol Procainamide	IV:5mg–10 mg (2.5 mg/kg) Oral: 600–2400 mg/d	21 days (31weeks + 3 days-34weeks + 3 Days)	Control	No side effects
Flack et al. (1993)	1993	Case report	1	SVT	1	No	Sotalol Flecainide	Oral: 200–600 mg/d IV: 15 mg (7 mg/kg)	3 weeks	Control	No side effects
Gembruch et al. (1989)	1989	Case report	1	SVT	1	No	Verapamil Disopyramide Propafenone Quinidine	IV: 10–40 mg Mother IV: 900–1200 mg/d	22 days	Control	No side effect No corneal deposits
Arnoux et al. (1987)	1987	Case report	1	SVT	1	No	Digoxin	Oral: 800–1,600 mg/d	6 weeks	Control	TSH abnormal
Laurent et al. (1987)	1987	Case report	1	SVT + CHF	1	No	Digoxin	Oral:1,200 mg/d for 3 days, then 600 mg/d for 3 weeks	3 weeks+3d	Improved	Hypothyroidism
Rey et al. (1985)	1985	Case report	1	SVT	1	No	Digoxin Propranolol Verapamil	Mother IV: 900 mg/d Oral:200–1,200 mg/d	3 weeks	Improved	No side effects
Moatassim et al. (2018)	2018	Case series	5	SVT	Not clear	Yes 1 No 4	Digoxin (5)	Oral: loading dose 1,200–1,600 mg/d, maintenance dose 400–600 mg/d	Not clear (until cardioversion)	Control 4	No side effects
Kang et al. (2015)	2015	Case series	8	SVT	8	Yes 1 No 7	Digoxin (3) Flecainide (7) Procainamide (1)	IP: a starting dose of 4–7 mg/kg (estimated foetal weight plus 25%) Oral: 200 mg tid IV	Once IP: 2 Twice IP: 1 4 times IP: 3 11times IP: 1 IV + IP: 1	Control 7	Transient hypothyroidism: 2
Pézard et al. (2008)	2008	Case series	14	SVT 8 AF 3 VT 1 AET 2	6	Yes 6 No 8	Digoxin (9) Verapamil (1) Flecainide (1)	Oral, loading dose: 1,600–2,400 mg/d, maintenance dose: 200–400 mg/d	Not clear	Control 12	TSH levels of elevated: 6 (2 with goiter)
Strasburger et al. (2004)	2004	Case series	26	AVRT 15 AF 9 VT 1 JET 1	9	Yes 2 No 24	Digoxin (25) Verapamil (5) Quinidine (5) Flecainide (4) Sotalol (2)	Oral, loading dose: 1800–2,400 mg/d, single doses not exceeding 800 mg	Loading dose 2–7 days, maintenance therapy 1–15 weeks	Control 19	Abnormal TSH or T4 levels: 5 Delayed hypothyroidism: 1, Mild language acquisition delay: 2
Jouannic et al. (2003)	2003	Case series	13	SVT	13	Yes 4 No 9	Digoxin (7) Flecainide (2) Sotalol (2)	Oral:loading dose of 1,600–2000 mg per day for 2 days and then reduced to 400–600 mg/d	between two and 8 weeks before birth	Control 7	mild hypothyroidism 2
Jouannic et al. (2002)	2002	Case series	7	SVT AF	0	Yes 6 No 1	Digoxin (2)	Oral: loading dose 1,600–2000 mg/d, maintenance dose 400–600 mg/d	Not clear	Control 3	Not mentioned
Simpson and Sharland (1998)	1998	Case series	3	SVT AF	3	No 3	Digoxin (2) Sotalol (1) Verapamil (1)	IV: 7.5–20 mg Oral 200 mg q8h IP	Not clear	Control 2	Not mentioned
Azancot-Benisty et al. (1992)	1992	Case series	5	SVT AF	2	Yes 0 No 5	Digoxin (5)	Oral: 800–1,200 mg/d	Not clear	Control 2	Hypothyroidism: 1
Hansmann et al. (1991)	1991	Case series	5	SVT AF	5	No 5	Digoxin (5) Propafenone Verapamil	(Mother) 1,200 mg i.v. as a loading dose for 5–7 days, 600–800 mg orally as a maintenance dose IV: 9–40 mg	IV: 7–15 times	Control 3 Improved 1 Died 1	No side effects 3 Transient hypothyroidism 1

CHF, cardiac heart failure; SVT, supraventricular tachycardia; AE, atrial ectopic tachycardia; VT, ventricular tachycardia; JET, junctional ectopic tachycardia; TSH, thyroid-stimulating hormone; AVRT, atrioventricular reciprocating tachycardia; AF, atrial flutter; IP, intraperitoneal injection; IV: intravascular injection.

### Qualitative synthesis of case reports

A descriptive statistical analysis was conducted on 21 fetuses with fetal tachycardia, derived from 18 case reports (Table 1; Table 2) (Adenin and Kapnosa Hasani, 2023; Huang and Wolf, 2019; Minisha et al., 2019; Lin et al., 2016; Capone et al., 2014; Pradhan et al., 2006; Khositseth et al., 2003; Vanbesien et al., 2001; Schleich et al., 2000; Mangione et al., 1999; Lupoglazoff et al., 1999; De Catte et al., 1994; Jouppila et al., 1993; Flack et al., 1993; Gembruch et al., 1989; Arnoux et al., 1987; Laurent et al., 1987; Rey et al., 1985). The cardioversion rate for amiodarone-inclusive protocol was 81.0% (17/21). SVT was diagnosed in 90.5% (19/21) of the cases, but all were accompanied by non-immune hydrops. Amiodarone was selected as the first-line treatment in only four of the 21 cases. Two pregnancies were managed with amiodarone monotherapy, of which one failed to control the fetal heart rate. Regarding dosage and administration, the most prevalent routes of amiodarone application in fetal tachycardia were transplacental transfer and direct injection. The oral doses of amiodarone varied from 100 to 2,400 mg/d, and the fetal IV dosage was between 2.5 and 40 mg, depending on the fetal weight. Moreover, the duration of amiodarone exposure was approximately 2–11 weeks. Of the 21 fetues in the cases, nine experienced amiodarone-related ADR (9/21, 42.9%), all of which presented as thyroid dysfunction, like abnormality of TSH or T4 level, and hypothyroidism. Although the incidence of adverse reactions to amiodarone was high in these case reports, it is important to consider that case reports tend to predominantly highlight positive outcomes and are often limited in the number of cases reported, which may introduce some bias in accuracy. Therefore, we further conducted a meta-analysis of the included case series.

**TABLE 2:** Summary of case reports on fetal tachyarrhythmia treated with amiodarone.

Author	Country	Cases no.	Hydrops	Amiodarone as first therapy	Transplacental transfer (oral)	Direct therapy (injection)	Fetal heart rate controlled?	Adverse drug reactions?
Adenin and Kapnosa Hasani (2023)	Indonesia	1	Y	Y	Y	Y	Y	N
Huang and Wolf (2019)	USA	1	Y	N	Y	N	N	Y
Minisha et al. (2019)	Qatar	1	Y	N	Y	N	Y	N
Lin et al. (2016)	Taiwan, China	1	Y	Y	N	Y	Y	N
Capone et al. (2014)	USA	1	Y	N	Y	Y	Y	Y
Pradhan et al. (2006)	India	1	Y	N	Y	N	Y	N
Khositseth et al. (2003)	USA	3	Y	Y (1)	Y (3)	Y (1)	Y (3)	Y (1) N (2)
Vanbesien et al. (2001)	Belgium	2	Y	N	Y (2)	Y (2)	Y (2)	Y (2)
Schleich et al. (2000)	France	1	Y	N	Y	N	Y	N
Mangione et al. (1999)	France	1	Y	N	Y	Y	Y	Y
Lupoglazoff et al. (1999)	France	1	Y	Y	Y	N	N	N
De Catte et al. (1994)	Belgium	1	Y	N	N	Y	Y	Y
Jouppila et al. (1993)	Finland	1	Y	N	Y	Y	Y	N
Flack et al. (1993)	UK	1	Y	N	Y	Y	Y	N
Gembruch et al. (1989)	German	1	Y	N	*M	Y	Y	N
Arnoux et al. (1987)	France	1	Y	N	Y	N	Y	Y
Laurent et al. (1987)	France	1	Y	N	Y	N	N	Y
Rey et al. (1985)	Canada	1	Y	N	Y	Y	N	N

Y. yes; N, no; *M, Mother IV.

### Quantitative synthesis with meta-analysis

**FIGURE 2:**
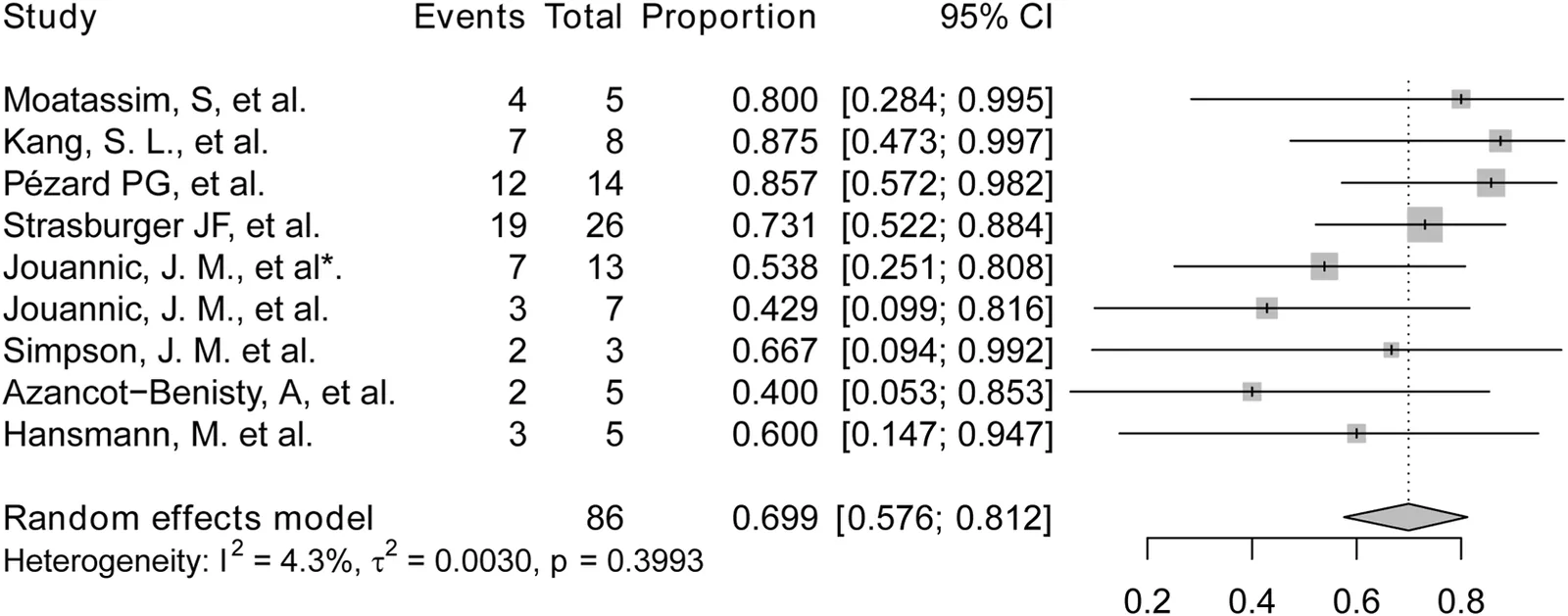
Forest plot of the pooled cardioversion rate for amiodarone-inclusive protocols in fetal tachycardia. *Used to distinguish two articles by the same author.

**FIGURE 3:**
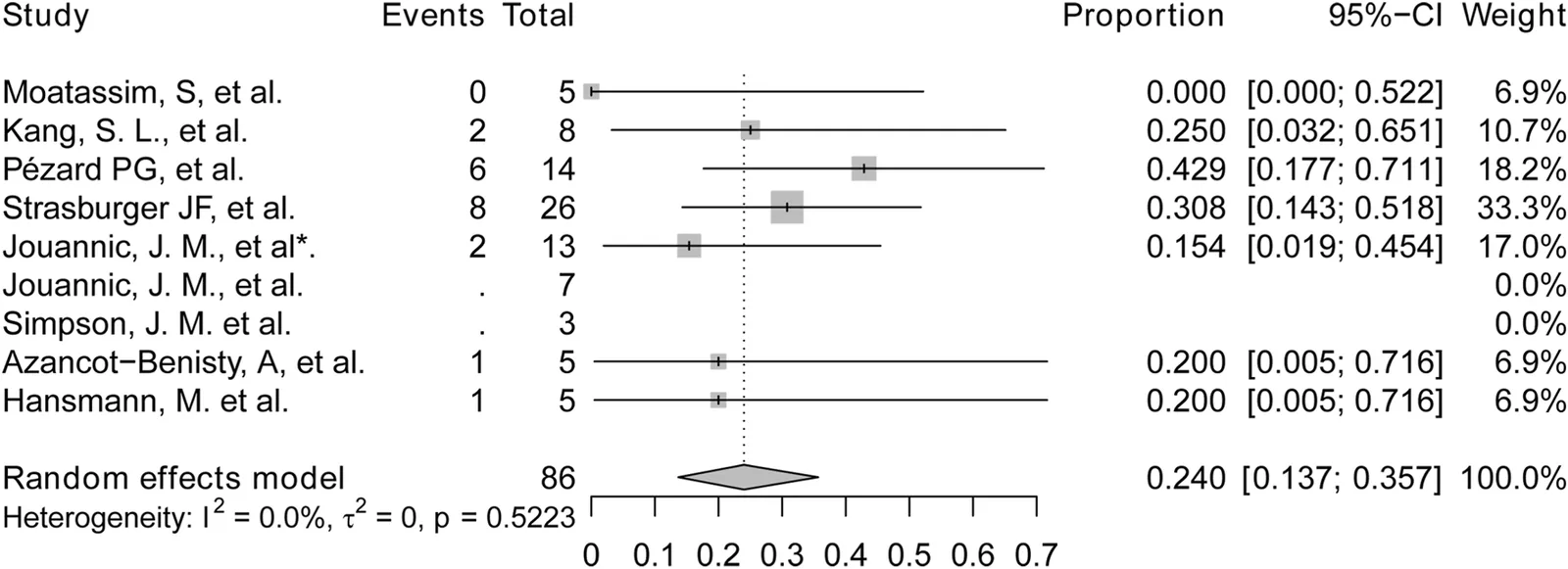
Forest plot of the pooled ADRs rate of amiodarone treatment in fetal tachycardia. *Used to distinguish two articles by the same author.

**FIGURE 4:**
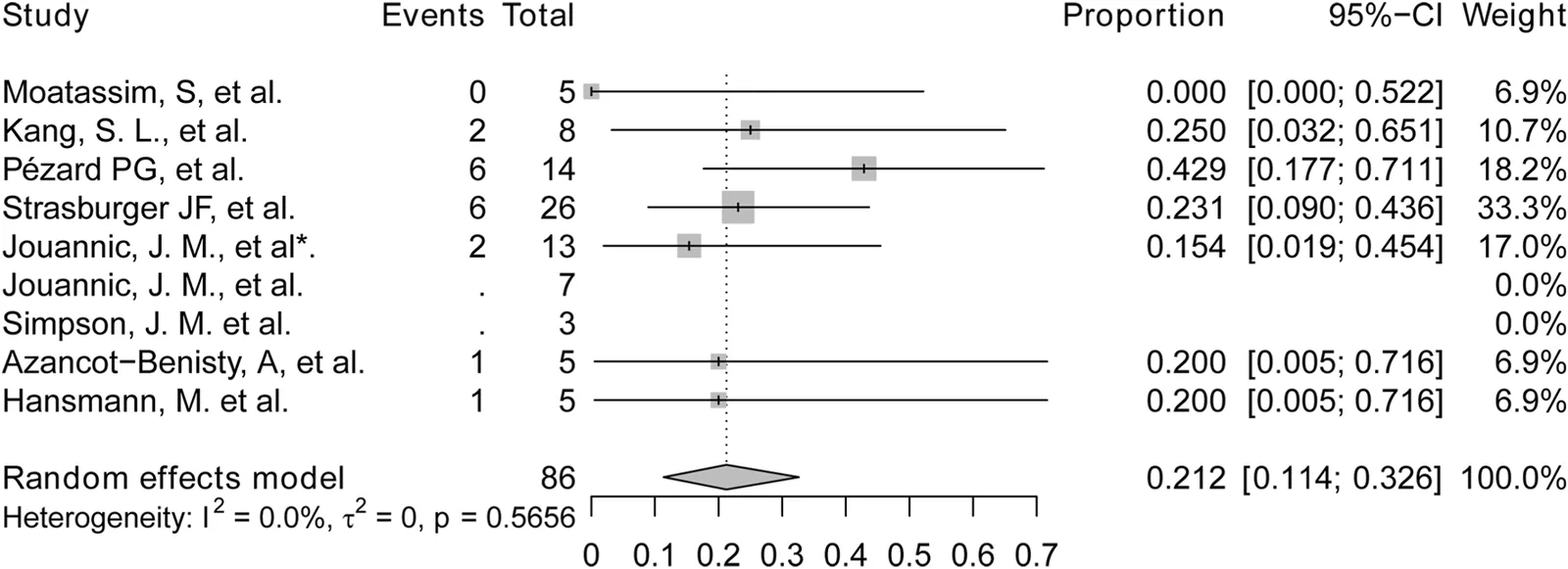
Forest plot of the pooled rate of thyroid dysfunction. *Used to distinguish two articles by the same author.

9 case series were included in this single-arm meta-analysis (Jouannic et al., 2002; Strasburger et al., 2004; Moatassim et al., 2018; Kang et al., 2015; Pézard et al., 2008; Jouannic et al., 2003; Simpson and Sharland, 1998; Azancot-Benisty et al., 1992; Hansmann et al., 1991). Amiodarone was administered to a total of 86 fetuses with fetal tachycardia, of which 59 pregnancies displayed rhythm conversion to SR or heart rate control. The proportion of fetus achieving cardioversion with amiodarone-containing therapy ranged from 40% to 87.5% across these nine studies (Table 3). A random-effects model with the Freeman-Tukey double arcsine transformation was subsequently performed to calculate the pooled cardioversion rate. The result showed a pooled cardioversion rate of 69.9% (95% CI: 57.6%–81.2%) with low heterogeneity (I^2^ = 4.3%, p = 0.40) for amiodarone-inclusive protocol (Figure 2). For safety outcomes, the present analysis examined the adverse reactions (ADRs) associated with amiodarone treatment for fetal tachycardia. Two studies (Jouannic et al., 2002; Simpson and Sharland, 1998) did not refer to safety outcomes and were therefore excluded from this analysis. Among 86 fetuses from seven studies, twenty developed ADRs following amiodarone exposure (Strasburger et al., 2004; Moatassim et al., 2018; Kang et al., 2015; Pézard et al., 2008; Jouannic et al., 2003; Azancot-Benisty et al., 1992; Hansmann et al., 1991) (Table 3), with a pooled rate of 23.98% (95% CI: 13.7%–35.7%, I^2^ = 0%, p = 0.52) (Figure 3). The most prevalent ADRs was thyroid dysfunction which occurred in 18 cases (90%), presented as abnormal TSH, T4 levels or mild hypothyroidism. As a result, the pooled incidence of thyroid dysfunction was 21.2% (95% CI: 11.4%–32.6%, I^2^ = 0%, p = 0.57), and there was no heterogeneity (Figure 4). All cases of thyroid dysfunction completely recovered following levothyroxine treatment. The remaining two infants had delayed language acquisition at birth, which subsequently normalized with age without any intervention (Strasburger et al., 2004). However, a causal relationship between amiodarone exposure and the speech delay in them could not be surely established.

**TABLE 3:** Summary of included studies for meta-analysis.

Author	Country	Hydrops	Total pregnancies with amiodarone	Cases of successful cardioversion	Effective rate (%)	Route of administration of amiodarone		Therapy		Cases of fetal adverse reactions	Cases of thyroid-related adverse events	Incidence of adverse reactions (%)
						Oral	Injection	Monotherapy	Combination			
Moatassim et al. (2018)	France	Not available	5	4	80.0	5	0	0	5	0	0	0
Kang et al. (2015)	UK	8	8	7	87.5	8	8	1	7	2	2	25.0
Pézard et al. (2008)	France	2	14	12	85.7	14	0	5	9	6	6	42.8
Strasburger et al. (2004)	USA	24	26	19	73.1	26	0	1	25	8	6	30.8
Jouannic et al. (2003)	France	13	13	7	53.8	13	0	Not available	Not available	2	2	15.4
Jouannic et al. (2002)	France	0	7	3	42.9	7	0	5	2	Not mentioned	Not available	Not available
Simpson and Sharland (1998)	UK	3	3	2	66.7	3	3	1	2	Not mentioned	Not available	Not available
Azancot-Benisty et al. (1992)	France	2	5	2	40.0	5	0	0	5	1	1	20.0
Hansmann et al. (1991)	Germany	5	5	3	60.0	3	5	3	2	1	1	20.0

### Descriptive subgroup analyses

Formal subgroup meta analysis were not performed owing to the limited number of studies and insufficient data. Subgroup analyses based on hydrops status, arrhythmia type, and route of administration were presented descriptively. Of the nine studies in the meta-analysis, four enrolled exclusively hydropic fetuses, with cardioversion rates ranging from 53.8% to 87.5% (median, 63.4%). A single study of non-hydropic fetuses reported a rate of 42.9% (Jouannic et al., 2002). Three studies included a mixed population with a cardioversion rate of 40%–85.7% (median, 73.1%). Six studies used oral amiodarone only (cardioversion rate 40%–85.7%, median 63.5%) (Table 3). Three research involved direct fetal injection (with or without oral administration), showing cardioversion rates of 60.0%, 66.7%, and 87.5% (median 66.7%) (Table 3). Regarding adverse events, five of the six studies reporting oral amiodarone administration showed adverse event rates ranging from 0% to 42.8% (median, 20%) (Table 3). Two studies combining oral and injection routes noted the ADR rates of 20.0% and 25.0% (Table 3), whereas the third similar study did not provide such data. Furthermore, all eight studies that reported the treatment included both amiodarone monotherapy and combination management. The proportion of monotherapy was 71.4% and 60% in the two studies, with corresponding cardioversion rates of 42.9% and 85.7%, respectively (Table 3). In the remaining six studies, the proportion of combination therapy ranged from 64.3% to 100% (median, 91.8%), with cardioversion rates of 40.0%–87.5% (median, 76.6%) (Table 3). Adverse event data were available in six of eight studies (0%–42.8%, median, 22.5%), since two studies did not report the safty outcome (Table 3).

### Risk of bias assessment

**FIGURE 5:**
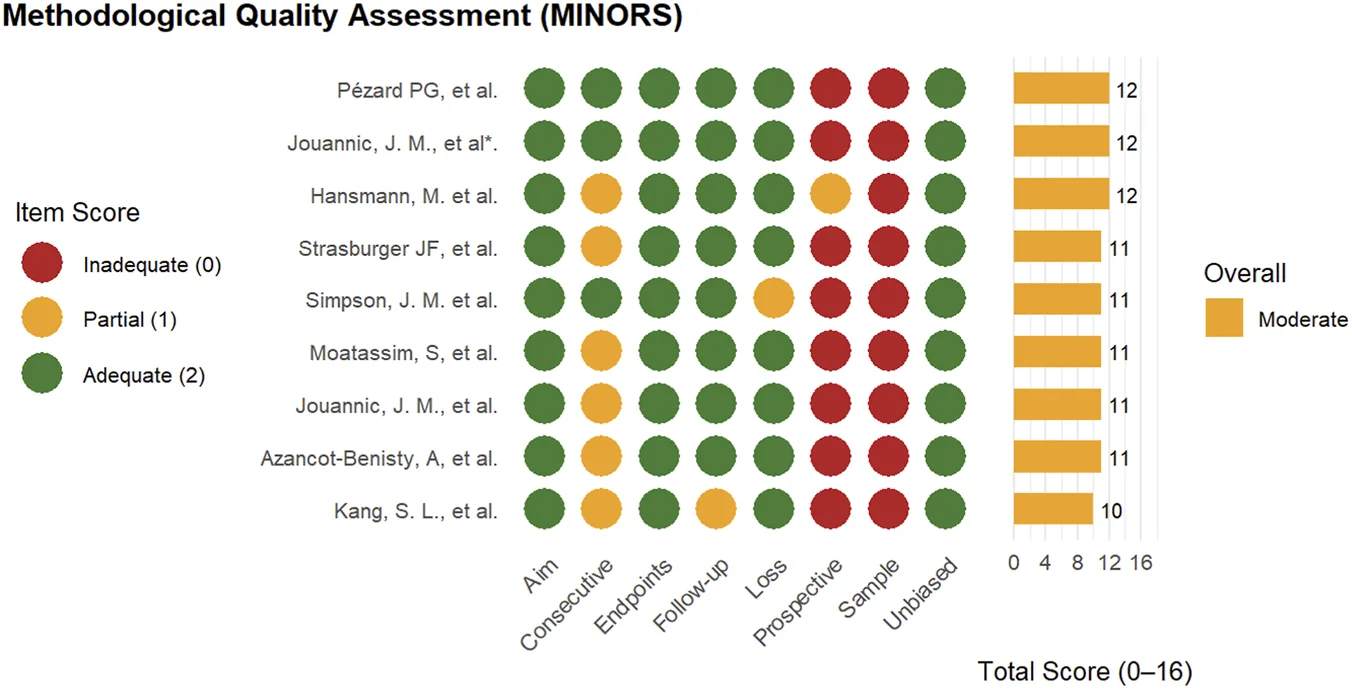
The risk of bias assessment of studies included in the single-arm meta-analysis. *Used to distinguish two articles by the same author.

Risk of bias for non-randomized studies was measured by the MINORS tool. All studies scored 11∼12 points (Table 4), indicating a moderate risk of bias (Figure 5). Case reports were evaluated via the JBI Critical Appraisal Checklist. All case reports received a score of at least 5, with 13 articles receiving a 7, one receiving a 5, as well as two articles scored 8 two scoring 6, respectively (Table 5).

**TABLE 4:** Quality assessment of the studies included in the meta-analysis.

Study	Q1	Q2	Q3	Q4	Q5	Q6	Q7	Q8	Quality score
Moatassim et al. (2018)	2	1	0	2	2	2	2	0	11
Kang et al. (2015)	2	1	0	2	2	1	2	0	10
Pézard et al. (2008)	2	2	0	2	2	2	2	0	12
Strasburger et al. (2004)	2	1	0	2	2	2	2	0	11
Jouannic et al. (2002)	2	1	0	2	2	2	2	0	11
Jouannic et al. (2003)	2	2	0	2	2	2	2	0	12
Simpson and Sharland (1998)	2	2	0	2	2	2	1	0	11
Azancot-Benisty et al. (1992)	2	1	0	2	2	2	2	0	11
Hansmann et al. (1991)	2	1	1	2	2	2	2	0	12

Numbers Q1-Q8 in heading signified.

Q1, A clearly stated aim.

Q2, Inclusion of consecutive patients.

Q3, Prospective collection of data.

Q4, Endpoints appropriate to the aim of the study.

Q5, Unbiased assessment of the study endpoint.

Q6, Follow-up period appropriate to the aim of the study.

Q7, Loss to follow up less than 5%.

Q8, Prospective calculation of the study size.

**TABLE 5:** Quality assessment of the case report.

Study	Q1	Q2	Q3	Q4	Q5	Q6	Q7	Q8	Quality score
Adenin and Kapnosa Hasani (2023)	1	1	1	1	1	1	1	1	8
Huang and Wolf (2019)	1	0	1	1	1	1	1	1	7
Minisha et al. (2019)	1	1	1	1	1	1	1	1	8
Lin et al. (2016)	1	0	1	1	1	1	1	1	7
Capone et al. (2014)	1	0	1	1	1	1	1	1	7
Pradhan et al. (2006)	1	0	1	1	1	1	1	1	7
Khositseth et al. (2003)	0	0	1	1	1	1	1	1	6
Vanbesien et al. (2001)	1	0	1	1	1	1	1	1	7
Schleich et al. (2000)	1	0	1	1	1	1	1	1	7
Mangione et al. (1999)	1	0	1	1	1	1	1	1	7
Lupoglazoff et al. (1999)	1	0	1	1	1	1	0	1	6
De Catte et al. (1994)	1	0	1	1	1	1	1	1	7
Jouppila et al. (1993)	1	0	1	1	1	1	1	1	7
Flack et al. (1993)	0	0	1	1	1	1	0	1	5
Gembruch et al. (1989)	1	0	1	1	1	1	1	1	7
Arnoux et al. (1987)	1	0	1	1	1	1	1	1	7
Laurent et al. (1987)	1	0	1	1	1	1	1	1	7
Rey et al. (1985)	1	0	1	1	1	1	1	1	7

Numbers Q1-Q8 in heading signified.

Q1, Were patient’s demographic characteristics clearly described?

Q2, Was the patient’s history clearly described and presented as a timeline?

Q3, Was the current clinical condition of the patient on presentation clearly described?

Q4, Were diagnostic tests or assessment methods and the results clearly described?

Q5, Was the intervention(s) or treatment procedure(s) clearly described?

Q6, Was the post-intervention clinical condition clearly described?

Q7, Were adverse events (harms) or unanticipated events identified and described?

Q8, Does the case report provide takeaway lessons?

### Sensitivity analysis

**FIGURE 6:**
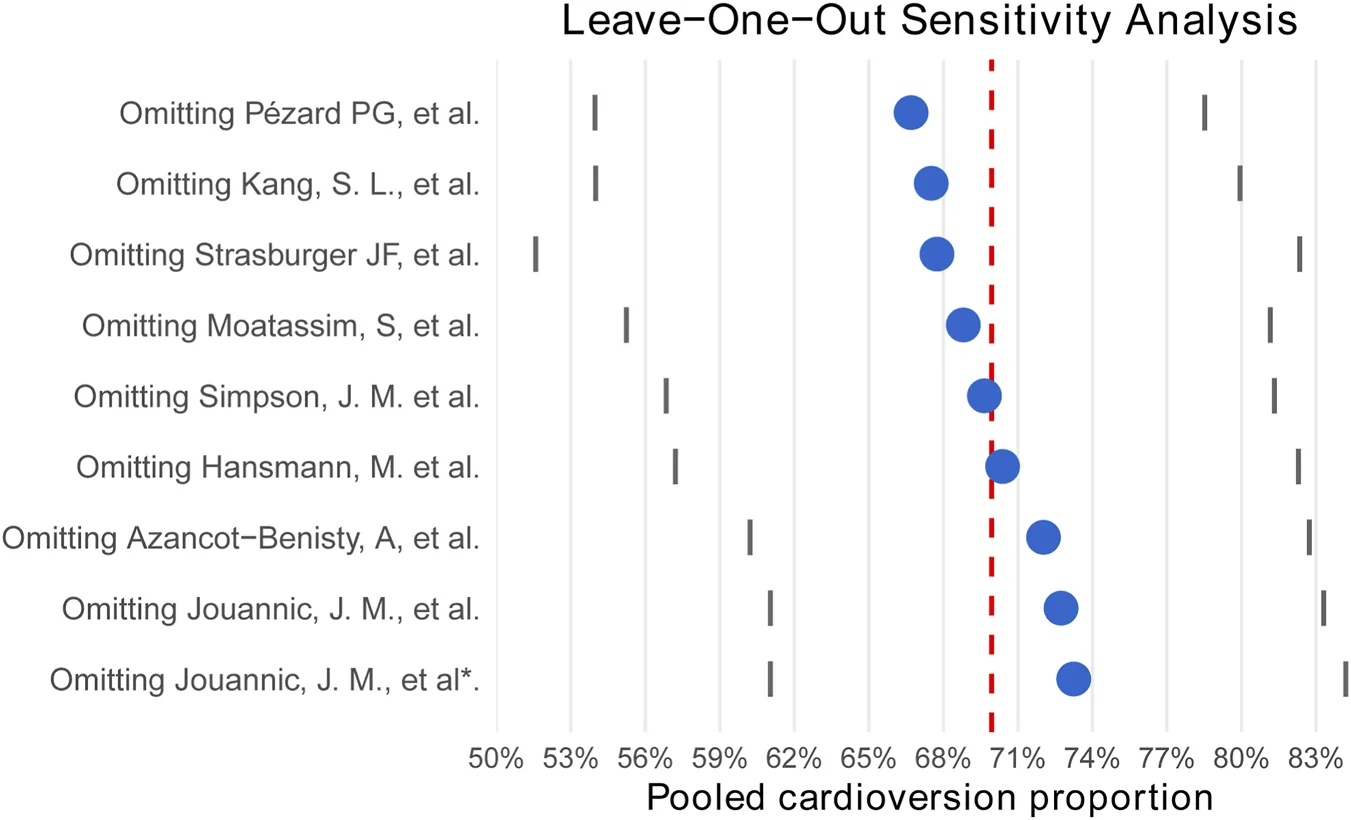
Leave-one-out sensitivity analysis of of the pooled cardioversion proportion. *Used to distinguish two articles by the same author.

Leave-one-out sensitivity analysis was conducted to verify the robustness of the pooled cardioversion proportion. The findings suggested that the exclusion of any single study did not significantly affect any of the summary results or their 95% confidence intervals. This indicated that the findings of our meta-analysis were solid and reliable (Figure 6).

### Publication bias

**FIGURE 7:**
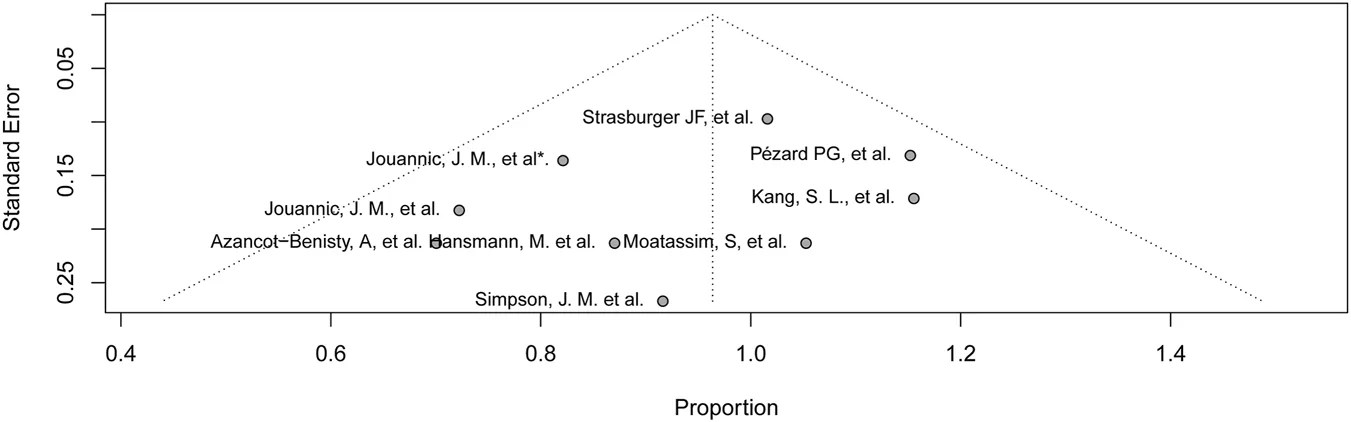
A funnel plot for publication bias test with 95% confidence limits of cardioversion rate. *Used to distinguish two articles by the same author.

The funnel plot showed a generally symmetrical scatter of studies, suggesting no strong indication of publication bias. However, this evaluation was mainly for reference since there were only nine studies (Figure 7).

## Discussion

This systematic review and meta-analysis mainly focused on the cardioversion rate of amiodarone-containing therapy for fetal heart rhythm, as well as adverse events in fetuses exposed to amiodarone. The descriptive synthesis part containing 18 case reports showed a cardioversion rate of 81.0%. However, this high rate is probably influenced by publication bias, as case reports tend to preferentially report positive outcomes. Moreover, the present findings reflect real world practice, where the most fetuses received amiodarone in combination with other regimens such as the digoxin. Accordingly, we acknowledged the observed efficacy as that of an amiodarone-inclusive protocol to ensure a more objective assessment. To improve the reliability of the findings, we performed a single-arm meta-analysis of 9 case series, which yielded a pooled cardioversion rate of 69.9% (95% CI: 57.6%–81.2%), suggesting a potentially beneficial role in achieving fetal cardioversion. This is comparable to previous reports on the effectiveness of amiodarone in severe, hydropic,and refractory fetal conditions (Gozar et al., 2022). Considering the factors influencing amiodarone treatment outcomes, we performed descriptive subgroup analyses of fetal hydrops status and administration routes as well as the combination therapy, according to the studies included in this meta-analysis. Four studies including hydropic fetuses all showed cardioversion rates above 50% (median, 63.4%), aligned with the overall pooled rate of 69.9%. Notably, in the mixed-study with the lowest overall rate (40%), the two fetuses that achieved cardioversion were both hydropic. These findings implied that amiodarone might exert a positive effect on the cardioversion of tachycardia, especially in severely hydropic fetuses. This may be attributed to the complex antiarrhythmic mechanisms of amiodarone and its active metabolites, as well as their delayed tissue deposition (Strasburger et al., 2004), despite poor transplacental transmission (fetal-maternal concentration ratio ranging from 0.1 to 0.28) (Alsaied et al., 2017; Hansmann et al., 1991).

Oral and injection-involved regimens were associated with median cardioversion rates 63.5% and 66.7%. Regretelly, these two groups which used different routes of administration are not suitable for a comparison of efficacy, because the baseline characteristics of the study populations were inconsistent. The fetuses receiving direct fetal injections were inherently more hemodynamically compromised or refractory than those treated orally. Direct injection of amiodarone may be advised as an alternative therapeutic approach for refractory fetal tachyarrhythmias, especially in fetuses with incomplete placental transfer (Lin et al., 2016; Kang et al., 2015; Hansmann et al., 1991). Nevertheless, it should be noted that direct injection is an invasive procedure associated with potential risks, including bleeding, amniotic fluid muddy, miscarriage, and even fetal cardiac arrest (Kunisaki and Jennings, 2008). Thus, we suggest that oral administration may be preferred as the initial approach because of its lower invasiveness. The use of amiodarone in combination was also described as a subgroup in the meta parts. Notably, all included studies contained both monotherapy and combination therapy, reflecting the pragmatic clinical approach of initial monotherapy with sequential escalation. In one study that included all combinations, with the lowest cardioversion rate (40%), the three non-responders all had cardiac failure, whereas the two responders presented with hydrops without failure (Hansmann et al., 1991). Similarly, the low prenatal conversion rate of 42.9% in the monotherapy-predominant study was due to treatment initiation after hemodynamic deterioration, manifesting as tricuspid regurgitation. Although these observations suggest that earlier treatment initiation before the development of severe myocardial compromise may be related to better outcomes, formal subgroup analysis stratified by exact timing of therapy initiation and degree of baseline myocardial compromise is not available. With this in mind, and considering the prevalent concomitant use of multiple agents across the included studies, amiodarone should be viewed as an adjunctive component of combination regimens rather than as a standalone monotherapy.

The safety of amiodarone for fetal development is still be a significant clinical concern. Historically, safety concerns regarding amiodarone have restricted its clinical use during pregnancy due to potential fetal risks (Strasburger et al., 2004; Batra et al., 2024). Nevertheless, the safety profile of amiodarone in fetal tachycardia management remains inadequate, with a particular paucity of evidence on its fetal impact. Among the 18 case reports included in our study, nine of 21 fetuses (42.9%) were associated with amiodarone adverse reactions, significantly higher than the 26.3% incidence rate derived from the 7 studies of our meta-analysis. However, potential reporting bias should be acknowledged. First, owing to publication factors, case reports with positive outcomes are more likely to be published, whereas those with negative findings are often unreported or unpublished. This could lead to an overestimation of adverse reactions in the analysis. Moreover, the limited number of studies coupled with the small sample sizes in both case reports and clinical studies may lead to inaccurate estimations of the incidence of adverse effects. Similar limitations were observed in our independent meta-analysis. The pooled rate of total adverse events was 23.98% (95% CI: 13.7%–35.7%), with thyroid dysfunction accounting for the vast majority (21.2%). The low statistical heterogeneity (I^2^ = 0%) across the seven studies lends consistency to this estimate. While the absence of heterogeneity (I^2^ = 0%) suggested consistent detection across studies, this apparent uniformity may reflect limited statistical power with only 86 fetuses, rather than true biological homogeneity. Importantly, most thyroid events were transient, only two neonates required short-term hormone replacement, and no cases of permanent hypothyroidism or other severe adverse events were reported. This provided evidence that the safety profile of amiodarone in this high-risk fetal population is dominated by reversible thyroid dysfunction. Therefore, this safety profile may be considered clinically acceptable in the absence of better alternatives. Nevertheless, the continued vigilance and postnatal thyroid monitoring of amiodarone remain warranted. Larger, prospective studies are needed to more precisely define its safety profile. We also considered whether the route of amiodarone administration would have different effects on fetal safety. There was no significant difference in the incidence of adverse reactions between oral (0%–42.8%, median 20%) and injection-oral modes of administration (20.0% and 25.0%).

In our studies, the oral dose of amiodarone for transplacental treatment ranged from 100 to 2,400 mg/d, while the direct injectable dose varied considerably (2.5–40 mg), generally administered at 2.5 mg/kg, depending on body weight. The duration of therapy was not uniform, varying from a minimum of 5 days to a maximum of 15 weeks. According to the AHA guidelines, the recommended maternal oral loading dose is 1.2–2.4 g/d for 2–7 days, followed by a maintenance dose of 0.2–0.4 g/d, consistent with the upper range of our observed doses. Notably, the lowest dose in our included studies is lower than the AHA maintenance recommendation, possibly reflecting clinicians’ caution regarding fetal safety given the limited evidence. The ESC guidelines mention amiodarone as an option for fetal tachyarrhythmia but do not provide specific dose recommendations, revealing the lack of standardized protocols for this rare condition. However, the occurrence of ADRs does not appear to be directly correlated with the dosage of treatment with amiodarone. Remarkably, healthy fetuses of mothers who suffer from arrhythmia were also exposed to amiodarone. Magee LA et al. reported thyroid dysfunction, neurologic abnormalities, and growth retardation in 12 such pregnancies following prolonged amiodarone exposure (≥15 weeks, including 7 with exposure throughout pregnancy) (Magee et al., 1995). This indicated that the risks associated with amiodarone might be correlated with exposure duration. Regarding the use of amiodarone in combination, eight of the studies included in the meta-analysis reported the use of amiodarone alone or in combination at length. Adverse event data were available in six of these eight studies (0%–42.8%, median 22.5%), but these six were not identical to the combination predominant group. Hence, no relationship between combination therapy and safety can be inferred. Further data are required to comprehensively evaluate the safety of amiodarone in fetal development.

According to the literature (Ross, 1998), the iodine contained in amiodarone poses a significant risk of adverse effects in the fetus. This is consistent with the findings of our meta-analysis, which showed an overall adverse event rate of 23.98%, with 21.2% of these events resulting from thyroid dysfunction. Amiodarone contains a large amount of iodine, approximately 37.5%, and undergoes deiodination in the body at a rate of 10% (Xanthos et al., 2007). Although direct iodine overdose in the fetus is rare, iodine can freely cross the placental barrier and accumulate in fetal tissues following exposure to amiodarone via various routes (Cohen-Lehman et al., 2010). Adults fully develop Wolff-Chaikoff effect, a protective mechanism that prevents excessive thyroid hormone synthesis induced by iodine overload, whereas this protective function in fetal thyroid does not mature until 36 weeks of gestation (Leung, 2012; Sohn et al., 2024). The thyroid of fetus begins to accumulate iodine at 10–12 weeks of gestation, therefore it is susceptible to be damaged from iodine overload between 10 and 36 weeks of pregnancy (Leung, 2012). As most fetuses in our analysis were exposed during this susceptible period, the observed 21.2% thyroid dysfunction rate likely explains the high proportion of ADRs. Consequently, the dosage of amiodarone should be carefully monitored when administered in before the final 4 weeks of gestation.

Given the lack of randomized trials of amiodarone in the fetus and the small sample size of the studies (Li et al., 2025; Pang et al., 2025), the results definitely have some limitations. An international, prospective, multicenter registry for fetal tachyarrhythmias is needed to contribute to the future guideline updates and optimize clinical care. Furthermore, fetal arrhythmia may be caused by various factors, including chromosomal abnormalities. Among all included studies, only one reported chromosomal evaluation without providing results (Mangione et al., 1999). Therefore, no meaningful subgroup analysis based on chromosomal abnormalities could be conducted, indicating that future studies that incorporate with routine genetic workup should be considered. In addition, it should be emphasized that these exploratory, hypothesis-generating findings derive from retrospective data and should not be interpreted as a challenge to the AHA guidelines, which position amiodarone as a third-line option.

## Conclusion

Summarily, amiodarone-inclusive protocol may exhibit a potential reversal effect in fetal arrhythmia, particularly in refractory condition. The primary adverse effect of amiodarone on the fetus is thyroid dysfunction. No reports of serious related adverse effects have been documented according to current research.

## Data Availability

The original contributions presented in the study are included in the article/supplementary material, further inquiries can be directed to the corresponding authors.
